# Immune Checkpoint Inhibitors and Cognition in Adults with Cancer: A Scoping Review

**DOI:** 10.3390/cancers17060928

**Published:** 2025-03-09

**Authors:** Síofra Hearne, Muireann McDonnell, Amanda Hanora Lavan, Andrew Davies

**Affiliations:** 1 School of Medicine, Trinity College Dublin, D02 PN40 Dublin, Ireland; 2 Mercer’s Institute for Successful Ageing, St. James’s Hospital, D08 NYH1 Dublin, Ireland; 3 Our Lady’s Hospice and Care Services, Harold’s Cross, D6W RY72 Dublin, Ireland; 4 Department of Medical Gerontology, Trinity College Dublin, D02 PN40 Dublin, Ireland; 5 School of Medicine, University College Dublin, D04 V1W8 Dublin, Ireland

**Keywords:** immunotherapy, immune checkpoint inhibitor, cognition, cancer

## Abstract

Cancer-related cognitive decline refers to a deterioration in cognitive function affecting adults with cancer at any stage of their cancer journey. Older adults are at increased risk of cognitive decline. As the indications for immune checkpoint inhibitors expand in the treatment of cancer, understanding the potential complicating cognitive issues experienced by those receiving this therapy will be important. The aim of this scoping review is to identify the literature regarding immune checkpoint inhibitors and subjective/objective decline, to identify evidence in older adults, differences between older and younger adults, and outline areas for further research. Four large electronic databases were searched. Records were screened using standardised methodology. Ten studies were identified that met the inclusion criteria for review. Six studies objectively evaluated cognitive function in adults receiving ICI treatment; eight studies performed subjective cognitive assessments. There were differences identified in the cognitive assessment tools used and the methodology between studies. Few studies reported on age-dependent findings. The results of this scoping review highlight the need for further research in this area using standardised methodology and testing, with a particular focus on the cognitive outcomes of older adults who may be at increased risk of developing cognitive decline while on treatment.

## Simple Summary

Cancer-related cognitive decline is a common problem. Many cancer treatments can contribute to problems with cognition. Immune checkpoint inhibitors have changed the face of cancer treatments in today’s world, yet the adverse effects of these treatments are still emerging, and little is understood about how they affect older adults. The impact of these treatments on cognitive function should be explored. This review aims to explore the existing research in this area. The findings of this review suggest that further research exploring the effects of immune checkpoint inhibitors on cognitive function should be undertaken. In particular, research methods and cognitive assessments used should be standardised. Furthermore, the cognitive effects of these treatments in older adults should be studied in greater detail.

## 1. Introduction

Cancer-Related Cognitive Decline (CRCD) refers to cognitive changes experienced by cancer patients at any stage of their illness journey [1]. It usually presents as a mild-to-moderate cognitive dysfunction affecting the domains of learning and memory, processing speed, and executive function [1]. Notably, subjective cognitive changes are common but do not always correlate with objective cognitive testing [2]. CRCD is understood to be multifactorial, involving complex and incompletely understood interactions between cancer, cancer treatments, and pre-disposing factors such as genetic factors and concomitant co-morbidities [3]. Indeed, most cancer treatments have been associated with cognitive decline, including surgery, radiotherapy, and systemic anti-cancer therapy (SACT) such as hormone therapy [4], chemotherapy [5], targeted therapy [6], and immunotherapy (see below).

Ageing is a risk factor for the development of cancer, with more than 60% of new cancer diagnoses occurring in persons >65 years old [7]. Cognitive impairment is common amongst older adults and may be related to a number of different aetiologies, including chronic and/or progressive conditions such as dementia [8] and acute, reversible conditions causing confusion and/or delirium (e.g., infection, metabolic problems) [9]. Unsurprisingly, older adults are at greater risk of developing CRCD, especially those with pre-existing low cognitive reserve, co-morbidities, psychological disorders, sleep problems, and frailty [10,11,12]. Nonetheless, the majority of the CRCD literature focuses on a younger adult population, particularly young women with breast cancer who have been/are being treated with systemic chemotherapy.

Immunotherapy has emerged as an important class of SACT over the last two decades [13,14]. Immunotherapy refers to therapies that utilise or enhance the body’s own immune system to target cancer cells [14]. Classes of immunotherapy include cytokines (e.g., interferons, interleukins), monoclonal antibodies (e.g., rituximab, trastuzumab), immune checkpoint inhibitors/ICIs (e.g., ipilimumab, pembrolizumab), cancer vaccines, and Chimeric Antigen Receptor-T (CAR-T) cell therapy [15]. There are important links between immune system regulation and normal brain functioning [16], and most of these classes have been linked with cognitive decline [17,18,19]. ICIs are licensed for use in many cancer types, including melanoma and non-small cell lung cancer [20]. ICIs target checkpoint proteins expressed on T-cells [21,22], ensuring ongoing activation of T-cells against cancer cells [22].

The aim of this scoping review was to review the literature regarding ICIs and subjective/objective cognitive decline: the objectives were to identify evidence in older (>70 years) adults, differences between older/younger patients, and areas for further research.

## 2. Materials and Methods

This review was conducted using standardised methodology [23,24,25]. The PRISMA extension for scoping reviews (PRISMA-ScR) was used to report this study [26]. A review protocol is registered at https://doi.org/10.17605/OSF.IO/N6XS2 (accessed on 26 January 2025).

### 2.1. Search Strategy

**Figure 1:**
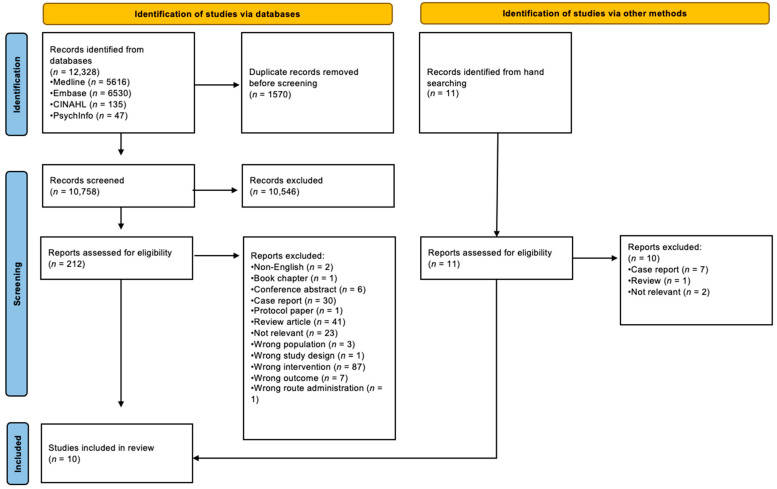
PRISMA ScR Flow Diagram.

Four electronic databases (OVID Medline, CINAHL, Embase, and PsycInfo) [Figure 1] were searched on 21st November 2024 using terms related to the domains of “cancer”, “immunotherapy”, and “cognition”. An example search strategy for OVID Medline is as follows: Immunotherapy.mp. or exp Immunotherapy OR immune checkpoint inhibitor.mp. or exp Immune Checkpoint Inhibitors OR programmed cell death 1 receptor.mp. or exp. Programmed Cell Death 1 Receptor OR programmed cell death 1 ligand 1.mp. OR CTLA-4 antigen.mp. or exp CTLA-4 Antigen OR lymphocyte activation gene 3 protein.mp. or exp. Lymphocyte Activation Gene 3 Protein OR exp Antineoplastic Agents, Immunological OR cancer immunotherapy.mp. OR exp Antibodies, Monoclonal/or monoclonal antibod*.mp. AND cognition.mp. or exp. Cognition OR exp Cognition Disorders OR cognitive defect.mp. OR cognitive reserve.mp. or exp Cognitive Reserve OR cognitive ageing. mp. or exp. Cognitive Ageing OR cognitive ageing.mp. OR exp Neuropsychological Tests OR exp Memory/or memory.mp. OR memory disorder.mp. or exp Memory Disorders OR exp Memory Consolidation OR cognitive impairment.mp. OR delirium.mp. or exp Delirium OR exp Dementia/or dementia.mp. OR disorders of higher cerebral function.mp. AND neoplasms.mp. or exp Neoplasms OR cancer*.mp. OR malignanc*.mp. OR tumour*.mp. OR oncolog*.mp. OR exp Medical Oncology.

The searches were not limited to any specific time period.

### 2.2. Study Eligibility Criteria

Eligible studies needed to include adult (>18 years) patients with non-primary CNS (central nervous system) cancer, who were receiving/had received ICIs, and who were undergoing subjective and/or objective assessment of cognition. Non-English language studies were excluded, as were conference abstracts, case reports, review articles, and other records without relevant original information.

### 2.3. Data Management and Synthesis

Article screening was performed utilising Covidence systematic review software (Veritas Health Innovation, Melbourne, Australia). Two reviewers (SH, AD) independently screened the titles and abstracts for full-text articles to review. A third reviewer (MM) was available to resolve potential conflicts relating to record inclusion. Two reviewers (SH, AD) independently reviewed the full-text articles and extracted the relevant information using a review-specific template. A third reviewer (MM) was available to resolve conflicts relating to data extraction.

The reference lists of all retrieved full-text articles were hand-searched for other relevant records.

Data were sought and reported on under the following headings: author, publication year, country, participant characteristics, cognitive assessment tools used, and outcomes relevant to the review.

## 3. Results

### 3.1. Search Results

The electronic database searches identified 10,758 unique references, while the “hand searching” identified another 11 unique references (see Figure 1). However, only 10 original studies met the inclusion criteria for the scoping review [27,28,29,30,31,32,33,34,35,36]. (see Table 1).

### 3.2. Studies Included

All included studies were observational: seven were longitudinal [27,28,29,32,33,34,35], and three were cross-sectional [30,31,36]. The median sample size was 43 (range 15 to 292). Six studies involved patients receiving ICIs [27,31,32,33,34,35], and four studies primarily involved patients who had received ICIs (“cancer survivors”) [28,29,30,36]. Two studies specifically investigated cognition in older (>70 yr) adults [31,35]. The other eight studies included older adults, but only two studies reported age-dependent findings [32,36]. No studies reported sex-related findings. Other study details are shown in Table 1.

### 3.3. Other Studies/Reports Identified

The search also identified one relevant ongoing study (the “Cog-Immuno trial”) [60] and a number of case reports of immune-related adverse effects (irAEs) resulting in cognitive impairment, e.g., seronegative encephalitis [61,62,63], autoimmune encephalitis [64,65,66], aseptic meningitis [67,68], cytokine release syndrome [69], hypothalamitis [70], and hypothyroidism [71]. Zhou et al. reported on ICI-related “psychiatric disorders recorded on the FDA Adverse Reporting System (FAERS) database (2012–2021) [72]: these accounted for 2.71% of adverse events, with the most common being “confusional state”, “delirium”, and “mental status change”. The risk of developing these adverse events was greater in older patients. Similarly, Kim et al. reported an increased incidence of delirium in patients treated with ICIs compared to patients treated with chemotherapy/targeted therapies within the South Korean population [73]. The mean age was 64.9 yr (SD +/− 10.0) in this study.

### 3.4. Objective Cognitive Assessment Tools Utilised

Six studies included an objective assessment of cognition, and 20 different assessment tools were utilised: (a) Montreal Cognitive Assessment (MoCA) [37]—used in two studies [27,33]; (b) MoCA-blind [49]—one study (in the remotely assessed patients) [33]; (c) Mini-Mental State Examination (MMSE) [43]—one study [31]; (d) Block design [56]—one study [33]; (e) Category fluency (animals and vegetables) [53]—one study [33]; (f) Cogstate test battery (multiple tests) [40]—two studies [28,29]; (g) Craft Story 21 Recall [50]—one study [33]; (h) Digit span forward and backward [51]—one study [33]; (i) Digit symbol [55]—one study [33]; (j) Immediate Hopkins Verbal Learning Test—Revised (HVLTi) [46]—one study [32]. (k) Delayed Hopkins Verbal Learning Test—Revised (HVLTd) [46]—one study [32]; (l) Letter number sequencing (LNS) [54]—one study [33]; (m) Stockings of Cambridge [47]—one study [32]; (n) Stroop Test [57]—one study [33]; (o) Test des neuf images-93 (TNI-93) [38]—one study [27]; (p) Trail Making Test (TMT) part A [45]—two studies [32,33]; (q) TMT part B [45]—one study [33]; (r) Oral TMT A [52]—one study (in the remotely assessed patients) [33]; (s) Oral TMT B [52]—one study (in the remotely assessed patients) [33]; and (t) Verbal fluency (F and L) [53]—one study [33]. It is important to note that the MoCA and MMSE are screening tools for mild cognitive impairment and dementia, respectively. The other assessment tools used are neuropsychological tests, which assess specific domains of cognitive function.

### 3.5. Subjective Cognitive Assessment Tools Utilised

Eight studies included a subjective assessment of cognition [28,29,30,32,33,34,35,36], and five different assessment tools were utilised: (a) the Cognitive Failure Questionnaire (CFQ) [42]—used in two studies [29,36]; (b) the cognitive function subscale of the EORTC-QLQ-C30—five studies [28,29,30,34,35]; (c) the Functional Assessment of Cancer Therapy-Cognitive Function (FACT-Cog) [44]—one study [32]; (d) the Patient Reported Outcomes Measurement Information System (PROMIS) Cognitive Function 8a [48]—one study [33]; and (e) the PROMIS Short Form Cognitive Function Abilities 8a [48]—one study [33]. Vanlaer et al. also undertook semi-structured interviews to assess patients’ subjective cognition [36].

### 3.6. Change in Objective Cognition During ICI Treatment

Three studies serially assessed objective cognitive function in patients receiving ICIs [27,32,33]: the reported frequency of objective cognitive impairment at baseline was 11.7–60% in these studies. In the largest study, Ma et al. [32] compared 240 matched pairs of patients with non-small cell lung cancer (NSCLC) and found statistically significant worsening of TMT (psychomotor speed, executive function), HVLTi (verbal memory), and HVLTd (delayed recall) scores at 6 months and 12 months in the ICI group versus the non-ICI group. However, only the TMT score at 12 months met the International Cognition and Cancer Task Force (ICCTF) criteria for cognitive impairment [74]. There was no evidence of worse results amongst older (>65 yr) patients. Another (small) study also reported worsening of cognitive function (i.e., MoCA-Blind but not other tests) [33], whilst the other (small) study reported stable cognitive function [27].

### 3.7. Change in Subjective Cognition During ICI Treatment

Three studies serially assessed subjective cognitive function in patients currently receiving ICIs [32,33,35]: one study reported worse baseline function (compared to the general population) [35], and two studies reported similar baseline function (compared to the control group/general population) [32,33]. In the largest study, Ma et al. [32] compared 292 matched pairs of patients with NSCLC and found statistically significant differences in “perceived cognitive decline events” (FACT-Cog scores) at 3 months, 6 months, 9 months, and 12 months in the ICI group versus the non-ICI group. There was evidence of worse results amongst older (>65 yr) patients. Myers et al. [33] reported no change in subjective cognition, but Suazo-Zepeda et al. [35] reported a “medium” change in subjective cognition in patients ≥70 yr (and trivial changes in younger patients).

Additionally, Jackson-Carroll et al. [75] performed a systematic review of quality of life in studies involving patients with advanced melanoma (*n* = 16) and determined that generally “patients exposed to ICI therapy were found to have stable (subjective) cognitive scores throughout the study that was similar to the scores in the compared control groups”. Similarly, Boutros et al. [76] performed a systematic review of health-related quality of life in randomised controlled trials comparing ICIs and chemotherapy and determined that patients receiving chemotherapy had greater (and quicker) worsening of subjective cognition than patients receiving immunotherapy [76].

### 3.8. Subjective Cognition in “Cancer Survivors” (Following ICI Treatment)

Four studies primarily involved patients who had received ICIs (“cancer survivors”) [28,29,30,36]. In three of the studies, participants completed the EORTC QLQ-C30 [28,29,30], and the cognitive functioning scale scores were statistically significantly worse than the normal population.

Additionally, Boekhout et al. [30] reported that 56% of participants experienced memory and concentration problems (data from the FACT-M questionnaire). Similarly, Vanlaer et al. [36] reported that 42.9% of participants reported memory and/or concentration problems (data from semi-structured interviews) and that these problems impacted their activities of daily living, including undertaking work-related tasks (14.3%), undertaking household tasks (17.1%), performing hobbies (10.0%), driving (4.3%), reading a book/newspaper (24.3%), and following a TV series/movie (8.6%).

### 3.9. Cognition in Older Persons (Versus Younger Persons)

Few of the studies reported on age-dependent findings. Ma et al. [32] reported statistically significant worse subjective cognition in those patients >65 yr receiving ICIs, although there was no difference in objective cognition between younger and older patients. Similarly, Suazo-Zepeda et al. [35] reported worse subjective cognition in those patients >70 yr receiving ICIs.

### 3.10. Subjective Cognition vs. Objective Cognition

Few of the studies reported on associations between subjective cognition and objective cognition. Ma et al. [32] reported that “there was a poor correlation between the outcomes of perceived cognitive impairment and objective neurocognitive tests” in patients receiving ICI treatment. Likewise, one study of cancer survivors reported “no significant correlations” [28], whilst another study of cancer survivors reported that there was a correlation between subjective cognition and a verbal memory test (but not the other cognitive tests) [29].

### 3.11. Cognitive Function and Specific ICI Therapies

Only eight studies reported the ICI received: the ICIs were nivolumab [27,32,33,34,36], pembrolizumab [28,32,33,36], ipilimumab [29,30,36], combination nivolumab + ipilimumab [36], and durvalumab [32]. No study reported differences in cognitive outcomes between ICIs.

## 4. Discussion

The review identified ten studies where cognition was a major endpoint, and there were significant differences in the populations (patients on treatment, “cancer survivors”), the assessment methods (subjective, objective), and the analysis/reporting of the data in the studies. Furthermore, many of the studies involved small numbers of patients and excluded specific cohorts of patients that regularly receive such treatment. Importantly, while many studies included older (>70 yr) patients, few studies reported separate results for younger/older patients.

Given the above, it is difficult to determine the exact impact of ICIs on cognitive function. Thus, further research is needed to answer the following unanswered questions:

(a) What are the short-term/long-term effects of ICIs on objective cognition (and what domains of cognition are affected)?
(b) What are the short-term/long-term effects of ICIs on subjective cognition?
(c) What factors predispose to ICI-related cognitive impairment (e.g., age, gender, comorbidities, pre-existing cognitive impairment, presence of cerebral metastases, ICI regimen, previous anticancer treatment)?
(d) Why is there limited concordance between subjective complaints and objective evidence of cognitive impairment?
(e) What is the underlying mechanism of ICI-related cognitive impairment?
(f) What are the optimal interventions for preventing/treating ICI-related cognitive impairment?

Furthermore, multicentre studies are needed in ICI-naïve patients, and these studies need to include baseline assessments of cognition (subjective, objective), periodic assessments during treatment, and ongoing assessments following treatment. [A number of the studies involved “cancer survivors”, and these studies suggest that patients that have previously received ICIs have ongoing (especially) subjective cognitive problems]. Further studies need to include “real world” patients and not exclude, for example, patients with pre-existing cognitive impairment or patients with cerebral metastases—previous studies have tended to exclude such patients. Moreover, further studies should utilise standardised/recommended neuropsychological assessments that address all of the domains of cognitive function and not just screening tools for cognitive impairment (e.g., MoCA). The latter will improve the quality of research studies and, importantly, facilitate the comparison of the results of different research studies (which is currently extremely challenging).

In 2011, the International Cognition and Cancer Task Force (ICCTF) recommended using the Hopkins Verbal Learning Test-Revised (HVLT-R), the Trail Making Test (TMT), and the Controlled Oral Word Association (COWA) of the Multilingual Aphasia Examination to assess cognitive function in patients with cancer—these assess learning and memory; processing speed; and executive function [74]. However, none of the review studies used all three tests, with one study using two tests (i.e., HVLT-R, TMT) [32], and one study using one test (i.e., TMT) [33]. Importantly, the ICCTF also made recommendations relating to study design, data analysis, and data interpretation: they suggested that cognitive impairment be established by “two or more test scores at or below –1·5 SDs from the normative mean (or the mean score of an appropriate control group), or a single test score at or below –2 SDs from the mean, or both”. Future research should incorporate these recommendations into methodology in order to harmonise research studies in this area and allow comparisons to be made between studies.

Nevertheless, despite all of the above limitations, there is evidence to suggest that ICIs are associated with cognitive impairment in some patients and that this may have a major impact on the quality of life of both patients on treatment and also those who have previously received treatment. Furthermore, while there is no firm evidence to support a mechanism by which ICIs contribute to cognitive decline, ICIs can cause various immune-related adverse effects (IrAEs), which can themselves contribute to cognitive dysfunction—including neurological IrAEs (e.g., encephalitis [77,78]) and systemic IrAEs (e.g., hypothyroidism [71]). Other, non-immune-related side effects such as neuropsychiatric presentations (delirium, insomnia, anxiety, and confusion [72]) and fatigue [79] could also potentially contribute to cognitive decline, particularly in older adults.

## 5. Conclusions

ICIs are a major advance in cancer treatment, although the impact of these treatments on certain aspects of patients’ quality of life, including cognitive function, still needs to be confirmed. Further research, utilising ICCTF recommendations on research methodology, is required to address these issues and to ensure that patients receive the best possible care.

## Figures and Tables

**Table 1:** Included Studies.

Study	Participants	Cognitive Function Assessments	Outcomes
Cuzzubbo et al., 2018 [27] France	*n* = 15 Median age-66 yr (range 33–86 yr) Female-8 Male-7 Melanoma-8 Non-small cell lung cancer (NSCLC)-7 Nivolumab-10 Pembrolizumab-4 Ipilimumab-1	Longitudinal study (assessments at baseline, 3 months) Montreal Cognitive Assessment/MoCA (evaluates executive function, visuo-spatial, short-term memory, working memory, language, attention, orientation) [37] “*Abnormal*” = *score* < *26*/*30* Test des neuf images-93/TNI-93 (episodic memory) [38] “*Abnormal*” *= score < 6 free recall or <9 total recall*	Baseline (*n* = 15): Abnormal MoCA—9 (60%) participants Abnormal TNI-93—5 (33.5%) participants “*Abnormal cognitive functions were associated with previous treatment with cytotoxic chemotherapy… and lung cancer*” 3 months (*n* = 9): Abnormal MoCA—6 (67%) participants (5 had abnormal MoCA at baseline) Abnormal TNI-93—3 (33%) participants (2 had abnormal TNI-93 at baseline) “*MoCA and TNI-93 scores were globally stable in the majority of patients*”
Rogiers et al., 2020a [28] Belgium	*n* = 25 Median age-58 yr (range 26–86 yr) Female-18 Male-7 Melanoma-25 Pembrolizumab-25 (12 on treatment, 13 post-treatment at baseline) “*Metastatic melanoma survivors*” “*Melanoma patients with unresectable AJCC stage III or IV disease were eligible… if they were on pembrolizumab treatment for at least 6 months and free from progression at their latest follow-up*”	Longitudinal study (assessments at baseline, 3–4 months, 5–7 months, 8–9 months, 10–12 months) EORTC Quality of Life Questionnaire-C30/EORTC QLQ-C30 cognitive functioning scale [39] Cogstate computerized battery of tests [40]: Detection test (processing speed); Identification test (attention); International Shopping List (verbal memory); delayed International Shopping List (verbal memory); One Back test (working memory); Groton Maze Learning Task (executive function) “*Impairment on a single test was classified when performance was lower than 1 standard deviation below normal age-appropriate mean*” “*Cognitive impairment was classified when abnormal performance occurred on at least 3 tests… in the battery*”	Baseline (*n* = 25): EORTC QLQ-C30 cognitive functioning scale—mean 75.0 (SD +/− 18.0) [Statistically significantly different from European reference values: *p* = 0.00025; European mean—90.5 (SD +/− 15.7) [41]] Cognitive impairment (Cogstate battery)—5 (20%) participants 3–4 months (*n* = 18): Cognitive impairment (Cogstate battery)—2 (11%) participants 5–7 months (*n* = 24): EORTC QLQ-C30 cognitive functioning scale—mean 76.4 (SD +/− 21.5) [Statistically significantly different from European reference values: *p* = 0.02] Cognitive impairment (Cogstate battery)—3 (13%) participants 8–9 months (*n* = 6): Cognitive impairment (Cogstate battery)—0 (0%) participants 10–12 months (*n* = 24): EORTC QLQ-C30 cognitive functioning scale—mean 79.9 (SD +/− 16.7) [Statistically significantly different from European reference values: *p* = 0.005] Cognitive impairment (Cogstate battery)—5 (21%) participants “*Performance was relatively stable across the five assessment timepoints… across each neurocognitive composite*” “*No significant correlations were found between memory, processing speed and executive function and… subjective cognitive function (EORTC QLQ-C30)*”
Rogiers et al., 2020b [29] Belgium	*n* = 17 Median age-63.4 yr (range 42–85 yr) Female-12 Male-5 Melanoma-17 Ipilimumab-17 (17 post-treatment at baseline) “*Metastatic melanoma survivors*” “*Eligible patients… unresect-able stage III or IV melanoma; survivors were disease-free for at least 2 years following start of IPI*”	Longitudinal study (assessments at baseline, 12 months) EORTC QLQ-C30 cognitive functioning scale [39] Cognitive Failures Questionnaire/CFQ [42] “*Impairment*” *= score ≥ 44* Cogstate computerized battery of tests [40]: Detection test (processing speed); Identification test (attention); International Shopping List (verbal memory); delayed International Shopping List (verbal memory); One Back test (working memory); Groton Maze Learning Task (executive function) “*Impairment on a single test was classified when performance was lower than 1 standard deviation below normal age-appropriate mean*” “*Impairment in NCF *(neurocognitive function)… *was classified when abnormal performance occurred on at least 3 tests… in the battery*”	Baseline (*n* = 17): EORTC QLQ-C30 cognitive functioning scale—mean 72.6 (SD +/− 27.6). [Not statistically significantly different from European reference values: *p* = 0.09; European mean—84.8 (SD +/− 21.3) [41]] Impairment CFQ—7 (41%) participants Impairment NCF (Cogstate battery)—7 (44%) participants (*n* = 16) 12 months (*n* = 15): EORTC QLQ-C30 cognitive functioning scale—mean 64.4 (SD +/− 25.9). [Statistically significantly different from European reference values: *p* = 0.009)] Impairment CFQ—7 (47%) participants Impairment NCF (Cogstate battery)—4 (33%) participants (*n* = 12) “*Only performance on the verbal memory test… was correlated significantly with ratings of… subjective cognition (CFQ)*”
Boekhout et al., 2021 [30] Belgium/Netherlands	*n* = 89 Median age-65 yr (range 23–87 yr) Female-38 Male-51 Melanoma-89 Ipilimumab-89 “*Advanced melanoma survivors*” “*Survivors eligible for this study… had survived at least 2 years following last admin-istration of ipilimumab for advanced melanoma… and were not diagnosed with recurrent systemic disease*”	Cross-sectional study EORTC QLQ-C30 cognitive functioning scale [39]	EORTC QLQ-C30 cognitive functioning scale—mean 83.7 (SD +/− 21.0) [Statistically significantly different from matched general population values: *p* = 0.001; matched general population mean—91.9 (SD +/− 14)] “*56% reported memory and concentration problems*” (data from Functional Assessment of Cancer Therapy-Melanoma/FACT-M questionnaire)
Invitto et al., 2022 [31] Italy	*n* = 43 Mean age-78 yr (SD +/−5.6) Female-10 Male-33 Cancer diagnosis-not stated Immunotherapy-not stated Control groups: “Oncogeriatric” chemo-therapy patients (*n* = 70) “Geriatric control” subjects (*n* = 41)	Cross-sectional study Mini-Mental State Examination/MMSE (spatial orientation, temporal orientation, memory skills, attention, calculus, language, constructive praxis) [43] “*Impairments*” *= < 22/30*	Immunotherapy group—mean MMSE score 23.942 (SD +/− 3.375) Chemotherapy group—mean MMSE score 24.276 (SD +/− 3.253) Geriatric controls group—mean MMSE score 25.127 (SD +/− 2.918) “*No differences related to the type of therapy emerged from MMSE*”
Ma et al., 2023 [32] China *Subjective data*	*n* = 292 (matched patients) Median age-62 yr (range 52–68 yr) Female-103 Male-189 NSCLC-292 Immunotherapy—not determinable Control group—matched NSCLC patients not scheduled to receive immunotherapy	Longitudinal study (assessments at baseline, 3 months, 6 months, 9 months, 12 months, 15 months) Functional Assessment of Cancer Therapy-Cognitive Function/FACT-Cog [44] “*Perceived cognitive decline events*” *(PCDE) = change in perceived cognitive impairment (PCI) subscore of > 0.5 SD mean baseline scores*	Baseline (*n* = 292 matched pairs): Mean PCI subscore—65.54 (SD +/− 11.49) immunotherapy group; 65.60 (SD +/− 9.34) control group 3 months (*n* = 292): PCDE—23 immunotherapy group; 1 control group (*p* < 0.001) 6 months (*n* = 292): PCDE—31 immunotherapy group; 3 control group (*p* < 0.001) 9 months (*n* = 292): PCDE—47 immunotherapy group; 8 control group (*p* < 0.001) 12 months (*n* = 292): PCDE—70 immunotherapy group; 7 control group (*p* < 0.001) 15 months (*n* = 292): PCDE—66 immunotherapy group; 9 control group (*p* < 0.001) “*Patients aged >65 years had significantly higher PCI score changes than patients aged ≤65 years (p < 0.01 for all sessions… )*” “*There were significant differences in* (PCI) *score changes… suggesting an increased level of cognitive decline as treatment progressed*” “*There was a poor correlation between the outcomes of perceived cognitive impairment and objective neurocognitive test*”
Ma et al., 2023 [32] China *Objective data*	*n* = 240 (matched patients) Mean age-61.08 yr (SD +/− 10.63) Female-86 Male-154 NSCLC-240 Nivolumab-113 Pembrolizumab-90 Durvalumab-37 Control group—matched NSCLC patients not scheduled to receive immunotherapy	Longitudinal study (assessments at baseline, 3 months, 6 months, 9 months, 12 months, 15 months) Trail Making Test/TMT A [45] Immediate Hopkins Verbal Learning Test—Revised/HVLTi [46] Delayed Hopkins Verbal Learning Test—Revised/HVLTd [46] Stockings of Cambridge [47] *Objective Cognitive Impairment (OCI) =* “*two test score changes ≥ 1.5 SD from baseline scores, or one test score ≥2 SD from baseline score*” *Cognitive Adverse Event (CoAE) =* “*any NBT* (neuropsychological battery test) score changes at each session that exceeded 3*SD of baseline scores”	Baseline (*n* = 240 matched pairs): OCI—28 (11.7%) immunotherapy group; 36 (15%) control group 6 months (*n* = 240): CoAE—82 (34.2%) immunotherapy group; 16 (6.7%) control group 12 months (*n* = 240): CoAE—102 (42.5%) immunotherapy group; 56 (23.3%) control group “*Objective deficits were observed in the 12 month in matched protocol TMT studies*” (but not other NBT scores) “*No significant difference… by age in NBT score changes*”
Myers et al., 2023 [33] United States America	*n* = 20 Median age-73.5 yr (range 32–88 yr) Female-8 Male-12 Melanoma-12 Other-8 Pembrolizumab-7 Nivolumab-6 Other-6	Longitudinal study (assessments at baseline, 6 months) Patient-Reported Outcomes Measurement Information System (PROMIS) Cognitive Function 8a [48]; PROMIS Cognitive Abilities 8a [48] MoCA/MoCA-Blind * (see above) [37,49] Craft story 21 Recall (episodic memory) [50] Digit span forward and backward (working memory) [51] TMT A/Oral TMT A * (processing speed) [52] TMT B/Oral TMT B * (executive function) [52] Verbal fluency (F and L) [53] Category fluency (animals and vegetables) [53] Letter number sequencing (working memory) [54] (Digit symbol) ** [55] (Block design) ** [56] (Stroop test) ** [57]	Baseline (*n* = 20): PROMIS Cognitive Function 8a mean T-Score 51.08 (SD +/− 8.86) PROMIS Cognitive Abilities 8a mean T-Score 54.51 (SD +/− 9.85) Estimated difference in MoCA-Blind score versus control data from “cognitively intact” persons −1.735 [95% CI: −3.591 to 0.122; *p* = 0.066] 6 months (*n* = 13): PROMIS Cognitive Function 8a mean T-Score 47.65 (SD +/− 8.07) PROMIS Cognitive Abilities 8a mean T-Score 51.62 (SD +/− 6.91) Change in PROMIS Cognitive Function 8a T score, and PROMIS Cognitive Abilities 8a mean T-Score, was not statistically significant Estimated difference in MoCA-Blind score versus control data from “cognitively intact” persons = −2.465 [95% CI: −4.304 to −0.627; *p* = 0.011] “*No significant within-group changes were noted for the CPI* (check point inhibitor) *group participants’ performances on the neurocognitive tests*”
Rogiers et al., 2023 [34] Belgium/Luxembourg	*n* = 125 (prospectively enrolled patients) Median age-60 yr (range 29–85 yr) Female-72 Male-80 Melanoma-152 Nivolumab-152	Longitudinal study (assessments at baseline, 9 months, 18 months—6 months post-therapy) EORTC QLQ-C30 cognitive functioning scale [39] “*Threshold for clinical importance*” *(TCI) = score < 75* [58]	Baseline (*n* = 125): EORTC QLQ-C30 cognitive functioning scale—mean 89.6 (SD +/− 19.7) [Not statistically significantly different from European reference values; European mean—84.8 (SD +/− 21.3) [41]] 17% patients score < TCI (*n* = 123) 3 months: 28% patients score < TCI (*n* = 95) 18 months: 34% patients score < TCI (*n* = 59) “*Although cognitive functioning mean scores remained stable, they decreased (deteriorated) and approached prespecified thresholds for MIDs* (minimally important differences)”
Suazo-Zepeda et al., 2023 [35] Netherlands	*n* = 151 Mean age-65.8 yr (SD +/− 9.25) Female-52 Male-99 NSCLC-151 Immunotherapy-not stated	Longitudinal study (assessments at baseline, 6 months) EORTC QLQ-C30 cognitive functioning scale [39]	Baseline: EORTC QLQ-C30 cognitive functioning scale <59 yr—mean 80.27 [Statistically significantly different from normative population value: *p* < 0.05; normative population mean—92.02 [59] EORTC QLQ-C30 cognitive functioning scale 60–69 yr—mean 86.11 [Statistically significantly different from normative population value: *p* < 0.05; normative population mean—92.90] EORTC QLQ-C30 cognitive functioning scale ≥70 yr—mean 88.71 [Not statistically significantly different from normative population value; normative population mean—90.10] 6 months: EORTC QLQ-C30 cognitive functioning scale <59 yr—mean 85.76 [Statistically significantly different from normative population value: *p* < 0.05] EORTC QLQ-C30 cognitive functioning scale 60–69 yr—mean 85.76 [Statistically significantly different from normative population value: *p* < 0.05] EORTC QLQ-C30 cognitive functioning scale ≥70 yr—mean 79.74 [Statistically significantly different from normative population value: *p* < 0.05] “*Older age (per 10-year increment) was negatively associated with change in… cognitive functioning*”
Vanlaer et al., 2024 [36] Belgium	*n* = 70 Median age-65 yr (range 34–92 yr) Female-28 Male-42 Melanoma-57 NSCLC-7 Other-6 Pembrolizumab-36 Ipilimumab/Nivolumab-13 Nivolumab-9 Ipilimumab-5 Ipilimumab/dendritic cell therapy-5 Other-2 “*Advanced cancer survivors*” “*Patients diagnosed with unresectable stage III/IV cancer of any type, who initiated ICB* (Immune Checkpoint Blockade) *at least one year prior… and who had a complete metabolic remission*”	Cross-sectional study Semi-structured interview CFQ [42] “*Moderate subjective cognitive complaints*” *= score ≥ 44* “*Severe subjective cognitive complaints*” *= score ≥ 55*	Interview—”*thirty patients (42.9%) reported having memory and/or concentration problems that impacted on their daily life activities*” (see text for further details) Moderate cognitive complaints CFQ—7 (10%) participants Severe cognitive complaints CFQ—6 (8.5%) participants Cognitive complaints were not correlated with age

* Used for remote assessments. ** Only used for face-to-face assessments.

## Data Availability

Data is contained within the article.
